# Anticholinergic agents and impaired cognitive function: is there a risk for patients with irritable bowel syndrome?

**DOI:** 10.1177/17562848251338479

**Published:** 2025-04-29

**Authors:** Peter J. Whorwell

**Affiliations:** Neurogastroenterology Unit, Wythenshawe Hospital, Manchester M23 9LT, UK

**Keywords:** anticholinergic, antimuscarinic, cognitive function, IBS

Anticholinergic medications have been used for therapeutic purposes since the 19th century, with atropine being the first representative of this class of drug. They are divided into antimuscarinic and antinicotinic subtypes, and today, there are three main therapeutic areas where the antimuscarinics are used. These are as follows: respiratory medicine for chronic obstructive pulmonary disease (COPD) and asthma, urology for the overactive bladder syndrome, and gastroenterology for abdominal pain and irritable bowel syndrome (IBS).

IBS is a common chronic disorder of gut–brain interaction,^1^ the principal symptoms of which are abdominal pain, bowel dysfunction and abdominal bloating or distension. However, patients also often complain of a variety of other non-colonic features such as low backache, lethargy, nausea and bladder symptoms.^2^ In the absence of a single effective treatment, ideal management should be holistic, involving educational, dietary, pharmacological and behavioural approaches using the biopsychosocial model of treatment. With regard to medication, there is no single agent that treats all the symptoms associated with IBS and, therefore, current management relies on targeting individual symptoms, with abdominal pain being regarded as a particular priority in the majority of sufferers.^3^

The cause of abdominal pain in IBS is thought to result from smooth muscle contractions (spasm), although there is some debate about whether these contractions are excessively strong and being sensed normally or of normal strength being sensed abnormally, possibly as a result of visceral hypersensitivity.^4^ Whatever the exact cause of the pain, the standard approach to treating it is to use medications that relax gastrointestinal smooth muscle (antispasmodics).

Antispasmodic medications are regarded as the first-line treatment of choice for managing the pain in patients with IBS.^4^ They are divided into two main classes: those with antimuscarinic activity (muscarinic receptor antagonists) and those that relax smooth muscle directly by a variety of mechanisms (anti-smooth muscle agents), and they have been available on prescription and over the counter in the UK for many years. However, as they were introduced long before the development of guidelines on the optimum design of clinical trials in general and IBS in particular, trials on the effectiveness of antispasmodics would not be regarded as ideal by modern standards. Despite this, meta-analyses of the available trials suggest a positive effect, and antispasmodics continue to be widely prescribed in both primary and secondary care as well as over the counter.^5^

Antispasmodics have always been considered to be extremely safe with little or no side effects, although antimuscarinics can cause a dry mouth and some blurring of vision, which is an inevitable consequence of their mechanism of action. However, more recently, the concept of anticholinergic burden has been developed based on concerns that muscarinic receptor antagonists may have the potential to accelerate cognitive decline, especially in older people, with the risk considered to be cumulative, irrespective of source, and dependent on total anticholinergic load resulting from all the medications that a patient is taking and the duration of exposure.^6–9^ There are a large number of commonly prescribed medications, such as the antihistamines and antidepressants, that have some degree of anticholinergic activity^10^ and physicians are being increasingly encouraged to review all the medications that a patient is taking to identify any possible risk. To aid this process and provide some form of quantification of anticholinergic burden, a variety of scoring systems have been developed that have been the subject of several reviews.^11–14^

This emerging concept of anticholinergic burden could lead to clinicians questioning the advisability of continuing to recommend anticholinergics for the treatment of conditions where they are currently traditionally used. This could result in patients being denied the benefits of a medication that has been keeping their symptoms under control for many years. The use of antimuscarinics in COPD and asthma should not be of concern, as the medication is typically delivered by inhalation, which reduces the chance of significant absorption. By contrast, antimuscarinics are taken orally in the overactive bladder syndrome and IBS, with the potential for systemic absorption and a negative effect on cognitive function. However, this effect is dependent on whether a drug is absorbed from the gastrointestinal tract and can penetrate the blood–brain barrier. In addition, the degree of affinity of a particular medication for muscarinic receptors in the brain would also be important.

With regard to their chemical structure, anticholinergics can be classified into tertiary amines (e.g., atropine, dicycloverine, hyoscine hydrobromide) and quaternary ammonium salts (e.g., hyoscine butylbromide and propantheline). Quaternary ammonium salts are highly polar, which results in them being poorly absorbed from the gastrointestinal tract and exhibiting poor penetration of the blood–brain barrier, in contrast to the tertiary amines. The availability of antispasmodics in general and anticholinergics in particular varies from country to country, and Table 1 lists some of the more commonly available anticholinergics on a worldwide basis and whether they have a quaternary ammonium structure.^15^

**Table 1.** Quaternary and non-quaternary ammonium antimuscarinic agents available in various countries around the world.

Quaternary ammonium antimuscarinics	Non-quaternary ammonium antimuscarinics
Hyoscine butylbromide	Hyoscine hydrobromide
Cimetropium bromide	Atropine
Glycopyrronium bromide	Hyoscyamine
Propantheline bromide	Trimebutine maleate*
	Dicyclomine (dicycloverine) hydrochloride

* Also a weak µ-opioid agonist.

Hyoscine butylbromide (Buscopan, butylscopolaminebromide) is a quaternary ammonium antimuscarinic medication which is extensively used in many countries for the treatment of IBS and is recommended in several guidelines.^5^ Its spasmolytic activity has been demonstrated in a number of mechanistic studies^16^ and its therapeutic potential confirmed in a meta-analysis of clinical trials involving patients with abdominal pain or IBS where it was shown to be the most effective spasmolytic.^5^ Approximately 8% of the drug is absorbed when it is taken by mouth, resulting in very low blood levels following therapeutic doses.^17^ Furthermore, it has been confirmed in animal and human ex vivo studies that hyoscine butylbromide in the gut lumen exerts its antispasmodic activity locally without the need for systemic absorption.^17,18^ The lack of systemic absorption significantly reduces the chances of any impairment of cognitive function, and the fact that hyoscine butylbromide is highly polar also results in poor penetration of the blood–brain barrier, making a negative effect on the brain even less likely.^17,19^ It should be noted that different hyoscine compounds, for instance hyoscine hydrobromide, have different pharmacological properties from hyoscine butylbromide, and this is not necessarily taken into account in some anticholinergic burden scoring systems.

IBS is a lifelong disorder the severity of which varies significantly from person to person. However, a sizeable proportion of sufferers experience daily abdominal pain requiring regular medication and spasmolytic agents are usually the first choice. Consequently, patients are likely to be advised to buy, or be prescribed, anticholinergics and this could be for many years, making the bioavailability of the antispasmodic concerned an important consideration. Consequently, anticholinergic burden needs to be taken into consideration, especially in elderly populations where multiple medications are often prescribed. Taken on its own, hyoscine butylbromide is extremely unlikely to contribute to this problem and, if taken with other medications with anticholinergic activity, will not significantly add to that burden. Therefore, hyoscine butylbromide can be considered a safe option for the long-term treatment of abdominal pain resulting from gastrointestinal spasm or IBS. Hyoscine butylbromide came to the market in 1952 when long-term safety studies were not required for new medications, although in its subsequent 75 years of widespread use, no significant safety concerns have ever emerged. It remains the most commonly prescribed antimuscarinic for IBS in the UK and is available in over 80 countries worldwide, comparing favourably with other antispasmodics in terms of cost. When effective, adherence to the medication is good, as side effects are seldom an issue.^16^

The standard dose of hyoscine butylbromide is 10–20 mg up to four times daily, but when the pain is particularly severe and only partially relieved by 20 mg, the dose can be safely increased, as it is likely that more than a local effect is needed and a degree of systemic absorption is required. Doses of 100 mg and 400 mg have been reported to be well tolerated^17,20^ and our practice is to gradually escalate the dose to 40, 60, 80, or even 100 mg at once or up to four times daily, as systemic absorption will still be modest, and penetration of the blood–brain barrier will still be impaired. Other ways that hyoscine butylbromide can be used include ‘as necessary use’ for intermittent abdominal pain, pre-emptive consumption before situations where pain might be anticipated, in conjunction with loperamide where pain control is required as well as control of diarrhoea, and in conjunction with a non-anticholinergic antispasmodic where pain control is suboptimal with the single medication.^16^

In the secondary care setting, hyoscine butylbromide can also be administered by intravenous or intramuscular injection. Most commonly, it is used intravenously to aid endoscopy when gastrointestinal contractions are causing problems, or in the emergency room or hospital when patients are suffering from severe abdominal pain.^21^ It can also be used effectively to prevent hospital admission in patients with recurrent severe abdominal pain who can learn to administer the medication intramuscularly themselves.^22^ Although the parenteral administration of hyoscine butylbromide bypasses the phenomenon of poor absorption from the gastrointestinal tract, there remains the safety net of poor penetration of the blood–brain barrier, especially as it is only used intermittently in this setting.

In conclusion, hyoscine butylbromide is an antimuscarinic medication that is a safe and effective spasmolytic agent. In contrast to many other drugs in the same class, it is poorly absorbed and fails to significantly penetrate the blood–brain barrier. Consequently, it is extremely unlikely to cause cognitive impairment, even if used on a long-term basis, and can be administered with other medications that have anticholinergic effects without adding to the anticholinergic burden. It could be argued that hyoscine butylbromide should not be included in any anticholinergic burden scoring system, but if it is, it should be given a very low score.

## Declarations

**Ethics approval and consent to participate:** Not applicable.

**Consent for publication:** Not applicable.

**Availability of data and materials:** Not applicable.
